# Book Reviews

**Published:** 1815-02

**Authors:** 


### CRITICAL ANALYSIS

### OF RECENT PUBLICATIONS

IN THE

### DIFFERENT BRANCHES OF PHYSIC, SURGERY, AND MEDICAL PHILOSOPHY.

*A Practical Treatise on Porrigo or Scalled Head, and on Impetigo, the humid or running Tetter; with coloured Engravings, illustrative of the Diseases.* By the late Robert Willan, M.D. F.R.S. and F.A.S. Edited by Ashby Smith, Member of the Royal College of Surgeons in London. 4to. Cox.

A PREFACE by the editor informs us, that the last period of Dr. Willan's life at Madeira was devoted to the task of revising his manuscripts. In this he was assisted by the editor, and, at the close of the doctor's earthly career, the papers were entrusted to the same hands.

It is afterwards added, that, in consequence of his near connection with the family, the editor's assistance was requested quested by Dr. W.'s representative in presenting the papers to the world.

"The foundation of Dr. Willan's whole fabric, consisting of a large portion of original notes, and all his case-books, are now in possession of the editor; these he has carefully consulted and compared; the terms of intimacy that subsisted between himself and the author, furnished him with ample means of ascertaining the extent of Dr. Willan's indefatigable zeal in all matters of medical research; and who, at length, contemplating the uncertainty of his recovery, unlocked to the editor, in moments of friendly intercourse, the vast stores of information which he had treasured up on various departments of medical science, but more especially on cutaneous disorders. It is from these opportunities of forming an opinion, that he has ventured to speak in favour of the present work.—The public, however, will form its own judgment.

"The greater part of the following pages is occupied with the description and treatment of Porrigo. The contagious property, and daily increasing extension, of this loathsome malady, render the investigation of it an object of peculiar interest; while the difficulty of removing it has long occasioned it to be classed among the *opprobria medicorum.* Indeed, from the time of the earliest medical writers, this branch of the healing art appears to have been involved in much obscurity; nor is the period in the history of medicine very remote, when the practice of it was, in a great degree, suffered to rest in the hands of empirics.

"How far this publication may supply the deficiency which has so long existed in the medical world, remains for the public to determine; but its utility, in arranging the varieties of scalled head, discriminating and illustrating their diagnostic symptoms, and recommending a mode of treatment, the result of thirty years' extensive and successful practice, will, it is conceived, be generally acknowledged.

"The latter part of this treatise contains a description of the appearances and treatment of Impetigo, the humid or running tetter; which, although the disorder possesses no feature in common with Porrigo, except its pustular origin, will be considered valuable, as the production of an author whose former labours have thrown so much light upon the subject of cutaneous diseases."

Thus, then, the case book was committed to Mr. A. Smith, and explained by frequent conversations. Such are the claims made by this gentleman to the mantle of Dr. Willan. Our readers may form their own judgment. For ourselves, we shall only remark, that the present work, by the closeness of the description, the accuracy of discrimination, the extended scale and minute delineation and chastity of colouring every where reminds us of Willan. We must also admit, that it is not without his failings; but these we can pardon where we see so much to commend.

The first and most important article is Porrigo, or Scalled Head. This term appears to us the most unobjectionable of any in use. It seems to have been the vernacular expression of the Romans, probably, at first, the same as prurigo or itching: it, like many other synonyms, acquired its appropriate meaning from necessity, as language acquired form and stability, and as health, in common with other enjoyments, became an object of closer and more accurate attention. The definition or character of the disease is not less unexceptionable.

"The porrigo is a contagious disease, without fever, usually exhibiting an eruption of the pustules termed Achores and Favi.

"The Achor is a small acuminated pustule, which contains a straw-coloured fluid, having the appearance, and nearly the consistence of strained honey. It appears most frequently about the head, and is succeeded by a dull white or yellowish scab. The Favus or Cerion is larger and less elevated than the Achor; being succeeded by a yellow, semi-transparent, and sometimes cellular scab, like a honeycomb."

Here we check ourselves from proceeding, having, in all our remarks on this learned author, regretted his frequent introduction of Galen, Actuarius, Rhazes, Trallian, Avenzoar, &c. &c. Yet even here we feel constrained to applaud the conclusion of this paragraph of the ancients:—"The word tinea," says our author, "has been often employed generally to express the different appearances of these pustular eruptions; however, as it belongs to the Arabic language, I declined the use of it in favour of the Latin term porrigo, which is familiar and appropriate."

Tinea is, perhaps, more common than porrigo in the present day. It is objectionable, not so much on account of its Arabic origin, as from the confusion it has made with the Latin term *tinea*, the tape-worm, or worms in general, so that at last we have got to the absurd distinction of tinea capitis, by the use of words from two remote countries, and having no meaning in common but as they both relate to disease. In a note to this passage, Dr. W. conceives the term porrigo derived from the odour of *porrum*, a leek. Vossius, however, imputes it to the involutions of a leek. Either of these or both may be right, or our former suggestion. But, as the decision cannot assist us in the designation of the disease, we have only introduced it to show to how little purpose hard names of authors and diseases are multiplied. It were well if this were the only objection. With the modest, they induce an apprehension of their own incapacity; with the confident, are the source of error; with the diligent, they consume that time which should be better employed; and they are little attended to by scholars or accurate practitioners.

tioners. In the present instance, we need no other proof of their inutility than the author's remark transcribed above, that, from the time of the earliest medical writers, this branch of the medical art appears to have been involved in much obscurity.

Under the genus porrigo, the author considers the following varieties: P. larvalis, P. furfurosa, P. lupinosa, P. scutellata, P. decalvans, and P. favosa.

P. larvalis is so called from the frequency with which it encrusts the whole face like a mask; it is the *crusta lactea* of the obstetric writers, a term which Dr. W. objects to, inasmuch as it is not always curable by a change of milk, nor in other respects necessarily connected with lactation, the following is the more general form of the disease.

"P. larvalis generally appears first on the forehead, in minute pustules, with a whitish point, set close together, and producing a redness and inequality of the surface, attended with considerable itching. The pustules break in a few days, and discharge a clear viscid humour, which gradually concretes into thin yellowish scabs. From beneath these a discharge of fluid takes place, from time to time, and forms additional layers of scabs, of a brown or blackish colour, till the forehead is completely incrusted. The scabs are in some places thick and rounded, though not very compact; in others, thin or laminated, and loose at the edges. They do not separate at regular periods; if any of them be detached, the surface is presently covered by a new incrustation. The scab is alternately dry and humid. Sometimes, from a fresh eruption of the pustules, or from other circumstances, the discharge becomes on a sudden so profuse that all the surface is laid bare, and remains for several days in a state of ulceration, emitting a thin, viscid, and acrimonious fluid from innumerable pores. Very young infants are most liable to be thus affected, and they suffer extremely from pain, itching, and irritation, when the complaint is extensive. On the cessation of the discharge, brown or blackish scabs gradually form again, and cover the ulcerated part. When the disease is about to terminate, the scab becomes dry, and sometimes whitish, and at length falls off, leaving a red shining cuticle, indented with deep lines, and very brittle,—hence it cracks and exfoliates, and is renewed, perhaps, three or four times before it acquires the usual colour and texture."

The complaint, however, appears in various parts of the head or face, and often spreads over the whole. The discharge has a disagreeable smell and is acrimonious. A prognostic of Dr. Strack's is mentioned of no inconsiderable importance, for whatever relates to prognosis is important, as the means by which the reputation of the practitioner is with great propriety decided. Dr. Strack says, that, when the complaint is disposed to terminate spontaneously, the urine urine acquires a smell similar to the urine of a cat. We wish we could learn whether Dr. Willan's observation confirms this remark.

On the treatment of the disease nothing particularly new occurs, excepting that the author seems less disposed to leave the case to nature than many of the older practitioners. His remedies are, ung. zinci, cerussæ, and hydrargyri nitrati, with the use of hydrargyri submurias, internally. Some other practical remarks follow in cases at all differing from the common, and last of all we have Professor Strack's specific, a decoction of viola tricolor in milk. The simplicity, and, if we may believe the professor, the certainty, of the remedy, and its speedy effect, make us again regret that our author gives us no hint concerning his own practice or even opinion on the subject. The editor, however, with much propriety informs us, that, as far as his knowledge extends, the doctor had not tried the remedy.

P. furfurosa is not to be considered a mere branny surface as the word would imply. "The eruption is from time to time renewed, the succeeding scabby incrustation is augmented, and often rendered soft by the exuding viscous fluid." The description is continued a few lines further, to distinguish it from the scaly leprosy, and some few other complaints which are dry and do not materially affect the head.

Dr. Willan found coculus indicus, as a local application, either sprinkling the powder or making it into a poultice, the most certain remedy. This was first recommended by Dr. John Hume in an inaugural dissertation, and afterwards adopted by Dr. James Hamilton, who communicated it as a remedy for Tinea Capitis to Dr. Willan.

Porrigo lupinosa consists of small clusters of achores. "These are formed in different parts, which presently break and form dry yellowish or white scabs. These within a fortnight gradually increase to the size of a sixpence, when they have a raised border, but are depressed towards the centre, and furrowed. There is often an intervening white incrustation, which nearly covers the scalp, and when dried, separates and falls off in small white laminæ. If the complaint be neglected, the occipital and submaxillary glands enlarge, and often suppurate, and the accumulating scab forms, with the hair, into a hard, elevated mass, sometimes resembling a bird's nest, or sometimes a pressed honeycomb.* A similar thick incrustation is formed in the Porrigo larvalis, affecting infants among the poor, when little attention is paid to neatness and cleanliness."

* "This appearance is presented in M. Alibert's fine prints, and in the Medical and Physical Journal, (London)."

Porrigo scutellata is only another word for the ring-worm of the head. The most useful part of this division relates to the probable origin and the infectious property of the disease, on both which we have some very judicious observations: for the treatment, we have a list of most of the well known remedies, with a remark, that it is sometimes necessary to try one, and sometimes another. As far as our own practice goes, however, we would say that the ung. hydrargyri albi has not failed in a single instance. By this, we would not be thought to question the difficulties which must sometimes have occurred to Dr. Willan in individual cases of his extensive practice.

The Porrigo decalvans, as may be supposed, is so named from its often destroying the hair. In these cases the scalp is smooth, shining in certain patches affected by the disease. At this passage of the work the author observes, that all the varieties may arise from the same contagion in the same manner as the various forms of scarlatina. If so, one should very much suspect, that the same remedy would be successful; and, perhaps, the only reason why it is not in all, may be certain stages of inveteracy, or degrees of inflammation, or habits of life. The first would only require a longer continuance of the remedy; the others internal remedies suited to the state of health or to the constitution. After this last observation, the reader will easily perceive that we have said enough for the purpose of a review. The learned remarks and quotations which follow, and the judicious application of certain remedies under particular circumstances, are well calculated for a book of reference, which Dr. Willan's work will for ever remain.

The next article in this book is on the Impetigo or running Tetter.

"By the term Impetigo," says our author, "I mean to express an eruption of the pustules usually denominated Psydracia. The Psydracium is a minute pustule, and irregularly circumscribed, producing but a slight elevation of the cuticle, and terminating in a laminated scab. Many of these pustules usually appear together, and become confluent. When mature, they contain pus; and, after breaking, discharge a thin watery humour."

Such a description is very concise, and seems intelligible; but the volley of quotation which follows, however it may impress us with a sense of the author's diligence and learning, certainly does not add to the perspicuity of the diagnosis. As, however, we have hitherto given no specimen of the author's learned citations, we shall, perhaps, not do justice to the work, if we omit the present opportunity.

"The definition of Psydracion by Greek authors, is conformable able to that given above. Alexander Trallianus and Paulus Ægineta represent Psydracia as small elevations of the cuticle, resembling Phlyctides, and chiefly confined to the head;* but, according to Galen, they appear on every part of the surface of the body, in a red papuliform eruption. Julius Pollux† has given the following definition: 'Psydracia are inflamed spontaneous eruptions (εξανθηματα) ulcerating superficially.'

"These pustules, when the eruption is extensive, do not appear uniform. Some of them are small and elevated; others larger, without a proportionate elevation of the cuticle; and a few of them resemble vesicles, not being filled with pus, but with a semi-transparent fluid.

"Celsus,‡ under the generic title Impetigo, has comprised several diseases which are not strictly connected with each other. The second species described by him, coincides with the Lepra or Psoriasis of the Greeks. The third and fourth are referable either to the Psoriasis inveterata, or to the scaly state of the skin in Elephantiasis. The first species appears to have been pustular, and may be ranked with the ulcerated Psora¶ of the Greeks. I take the liberty of employing the term Impetigo to express this species only, having, in my arrangement, separated pustular from scaly diseases of the skin, and having elsewhere described, under other denominations, the scaly complaints mentioned by Celsus. His description of the pustular impetigo is very short: 'Minimè mala est quæ similitudine Scabiem repræsentat, nam et rubet, et durior est, et exulcerata, et rodit. Videnturque esse in eâ quasi bullulæ quædam, ex quibus, interposito tempore, quasi squamulæ solvuntur.' He distinguishes this species from another, which is more ulcerated, and exhibits larger pustules resembling Vari, probably referring to Ecthyma, a disease characterised by large inflamed pustules, and not without affinity to the impetigo. His

---

"* Ψυδρακια εισι μικραι υπεροχαι, φλυκτισιν ομοιαι, υπερκειμεναι της επιφανειας.

Alex. Trall. lib. i. cap. 5. Paul. Æg. lib. iii. cap. 3.

"Ψυδρακιον εστι κοινως λεγομενον ὁ περι παν το σωμα, και περι το λευκον τȣ οφθαλμȣ γινεται, οιον εξανθημα εξ ακρȣ ερευθες.

Galen Introductio, cap. 15.

"† Ψυδρακια, πυρωδη εξανθηματα αυτοματα, ελκωδη εν επιφανεια.

Jul. Poll. Onomasticon, cap. 25.

"‡ De Medicin. lib. v. cap. 28. § 17.

"¶ On Cutaneous Diseases, p. 148. The Arabian physicians, in like manner, constitute a dry and a moist Alkouba or Impetigo. 'There is a sanguineous Kouba, which discharges a dewy moisture upon being rubbed.' Avicenna, vol. ii. p. 249. Compare Serapion, Breviar. Tr. v. cap. 2. Alsaharav. Tr. xxxi. cap. 7. Forest. Obs. Ch. lib. ii. p. 168. Hoffman, tom. iv. p. 438. and Supplem. 1. p. 143."

additional additional observation, that the latter occurs periodically, or with more regular intervals than the former, is also strictly true."

The divisions of this genus are, Impetigo sparsa, I. figurata, I. erysipelatodes, I. scabida, and I. rodens. A description of the first will enable us to give the reader a sufficient knowledge of the rest, as far as can be conveyed without consulting the work. In this instance, too, the plates are a very great assistance.

"In the Impetigo sparsa, the pustules are at a distance from each other; and the eruption extends, without any certain order, along the backs of the hands, the arms, neck, shoulders, thighs, or legs. After a few days, the pustules break, and discharge a thin humour, which gradually concretes into yellowish laminated scabs. The cuticle, as far as the eruption extends, becomes reddish, rough, or scaly; and a slight discharge from rhagades or chops in various places, as well as from beneath the thin scabs, continues through the complaint: the duration of which, in the upper extremities, is seldom more than two or three weeks. When the lower extremities are affected with this eruption, it continues a long time. Small yellow pustules first appear on the instep, and then on the ancle and leg, with a violent itching. They are most numerous on the foot and ancle, and when they are broken, a considerable quantity of humour issues from small pores, around which the cuticle is rough, reddish, shining, and a little elevated. The parts affected are for some weeks covered with thin scabs, but not sufficiently so to prevent the watery discharge. When the surface appears to be healed, and the scabs are about to separate, a fresh eruption of pustules often takes place, and the discharge recommences with great heat and irritation. After several returns of the eruption, ulcers are sometimes formed on the fore part or sides of the ancle. The ulcerations discharge a clear ichor; they exhibit a considerable but unequal cavity, and irregular edges surrounded by the pustules. In sedentary persons, who have passed the middle period of life, the edges of the ulcers are blackish, or of a purple hue, and the limbs become œdematous. The small pustules diffused over the surface are of nearly the same colour, and sometimes the intervening skin appears livid, or speckled with livid and red.

"The Impetigo sparsa is most troublesome when the yellow Psydracia are intermixed with small irregular vesicles, as frequently happens on the upper extremities. The complaint commences about the knuckles, and spreads along the thumb and fingers to the nails; likewise along the back of the hand, and round the wrists, to the fore-arm. Both hands are usually thus affected about the same time, and the eruption extends, in some cases, to the bend of the elbows, the upper arm, the neck, and the cheek. It is always succeeded by a little watery discharge, and by the formation of laminated scabs: when these fall off, the cuticle beneath remains for a long time scaly, and chopped, and in this state of it, fresh fresh pustules arise, with heat, soreness, and violent tingling. Thus, by repeated suppuration, and scabbing, the texture of the skin becomes, in many places, rough, harsh, and inflexible."

Impetigo figurata differs from the *sparsa* principally in the form of the eruption, the psydracia being so clustered as to form large scabs. In this respect, it resembles the ring-worm described by some tropical writers.

I. erysipelatodes partakes of the tumour and disposition to cuticular elevations which characterizes erysipelas: the elevations containing an ichorous instead of serous fluid.

The scabida and rodens are the most serious forms of the disease. The first includes that incrustation resembling the bark of a tree, which sometimes envelopes the legs of old subjects, and, without seeming to shorten life, renders it extremely irksome. The second is not confined to the surface, but affects the cellular membrane. Both these are described with much accuracy; and, for the last, the author seems fearful of offering any remedy, remarking that he never saw it terminate but with life. In most cases he has found it supervene a general ill state of health. It is probable, that the doctor's celebrity may have introduced him to many of these cases in their worst form, or in the inveterate stage; in which case, it is certain, but little can be done. On this account, we could wish the caution against such a progressive increase had been most pointedly marked. In the early stage only, can any thing be attempted for these complaints: in the more advanced, the I. *scabida* is often not less incurable than the *rodens*, especially in old subjects, whom it chiefly affects.

For the three first forms, Dr. W. recommends frequent washing the parts, occasional purges, and, in some cases, the Peruvian bark with the muriatic acid; as local applications, when necessary, the zinc and saturnine ointments. In the tropical ring-worm, however, the treatment is much more severe: lime-juice, gun-powder, and ketchup, are frequent remedies for old local complaints in the West Indies, the object of which can only be by a strong stimulus to induce a new action on the parts. I. erysipelatodes requires the mixed treatment which must be governed by the degree of inflammation; for the *scabida*, the mode of treatment here recommended may be very judicious if this form of the disease attacks young or middle-aged subjects. But, as we before remarked, inveterate cases in old subjects have, to us, all proved refractory. Both of them may be much mitigated in their early stage, if the health is tolerable or can be restored. The progress of the disease should be carefully watched, and treated with antiphlogistic remedies as often as the symptoms of the constitution seem to threaten a first paroxysm, roxysm. The local treatment must be varied according to the stage of the disease, but it is never desirable to leave the issue to the uninterrupted efforts of nature.

Such are the outlines of this valuable posthumous morsel. Having, in the progress of our review, already given an opinion of its merits, it cannot be necessary to add more. To recommend it may seem superfluous, as none who are in possession of the doctor's former numbers will fail to add this. But we would further remark, that, independent of every such consideration, the present sheets make a complete and valuable whole on diseases, the terror of parents who have children even at the best regulated schools, hitherto most imperfectly described and too often empirically treated. The editor has been careful to give an explanation of certain terms, without referring to the early definitions.

---

*The Case of Joanna Southcott, as far as it came under his professional Observation, impartially stated.* By P. MATTHIAS, Surgeon and Apothecary, Mableton-place. 8vo. 1s. pp. 22. Callow.

*A correct Statement of the Circumstances that attended the last Illness and Death of Mrs. Southcott, with an Account of the Appearances exhibited on Dissection, and the Artifices that were employed to deceive her Medical Attendants.* By RICHARD REECE, M.D. 8vo. 4s. pp. 107. Sherwood and Co.

IT appears that Mr. Matthias was first consulted upon Mrs. Southcott's case, by Mrs. Townly, whom he was then attending, and with whom the prophetess resided. Without being permitted a sight, enquiries were made concerning the probability of a virgin of sixty-four being pregnant, to which, of course, a rational practitioner could make only one answer. In the month of August last, however, he was admitted to the august presence of this celebrated female, and even allowed, among other examinations, *pectora tractare*. This is the only part of the examination which we shall take the trouble of noticing.

Mr. Matthias says, "I could find no appearance of milk in her breasts: they were swollen, and the vessels full and tumefied, but more like glandular enlargements." Now, if the breasts were enlarged from pregnancy, what could that increase consist of but glandular enlargement, with full and tumefied vessels? No notice is taken of the appearance of the nipples or areolæ. It is remarkable that all who give any account of this case, admit some change in the mammæ, which is a fact worth recording in a woman of that age, and of which we shall hereafter say more.

Mr. M. seems, according to some very prevalent opinions at present, to impute most diseases to the biliary system. Thus the colour of the stools is carefully described, and they are said to be sometimes clay-coloured, sometimes black and offensive; and, among other remedies, we find *hydrargyrus submuriatus* proposed. But, like some others, Mr. M. was soon dismissed, when it was found that his opinion did not accord with the wishes of Joanna and her friends.

Dr. Reece, though he did not see the patient till a most respectable physician had pronounced that she was not pregnant, did not scruple to give a different opinion. He was introduced by one of her believers, a most pious man, and afterwards waited on by a clergyman of the English church, a near relation of a noble lord, whose name he bears, and several others; of all whom we may say, that they were anxious to be deceived. The rest of the detail is too uninteresting and too generally known to require our notice. We shall only offer a few remarks on the dissection.

Nothing seems to have occurred worth notice but the enormous quantity of fat. There is some obscurity in the account of the liver. Dr. Reece says, "The liver appeared dark, and rather soft in its texture, which I considered to be the consequence of a disorganizing process taking place after death; it was not enlarged." Mr. Matthias says, "The liver appeared as one putrid mass, but of its natural size." One putrid mass is very different from a dark appearance, and rather soft in its texture. However, as the whole body was putrid, we would make allowance for the different language of different writers. But, in accounting for the symptoms during life, Mr. M. adds, "The affection in the kidneys, various pains in the loins and shoulder-blade, are ascribable to this organic derangement of the liver and gall-bladder."

We have dwelt so long on this apparently-minute circumstance, to show how possible it is for the best-intentioned people to render prominent a fact of little importance. All the other medical people whom we have seen, agree that the liver appeared sound; but the necessity of hydrargyrus muriatus in so many diseases, and all for the purpose of curing the liver, has induced a too general opinion that all are ascribable to that cause. It is true, gall-stones were found in the gall-bladder, but they do not appear to have obstructed the passage of the gall.

Dr. Reece conceives the circumscribed enlargement in the lower part of the abdomen, and which he perceived during life, life, arose from the contrivance of the deceased to keep the urinary bladder full, when she expected to be examined by one of the faculty. This, we conceive, could hardly have been perceived through so large a quantity of fat. The altered appearance of her breasts, and even of her nipples, he imputes to the artificial extraction of the latter.

On a retrospect, the whole appears perfectly ridiculous; but this short notice may at least serve as a memorial of an event which at one time produced no inconsiderable effect on the public mind. It may not be destitute of another advantage. Joanna swore herself with child. It is not uncommon for younger women to swear themselves not with child. The uncertainty of any examination excepting *per vaginam*, should at least make us cautious of a hasty decision, however suspicious the symptoms may seem.

---

*The Morbid Anatomy of the Brain in Mania and Hydrophobia, with the Pathology of these two Diseases, as collected from the Papers of the late Andrew Marshal, M.D. &c.* By S. SAWREY, Member of the Royal College of Surgeons, &c.

(*Concluded from p.* 60.)

IN our last we gave the most interesting particulars of Dr. Marshal's life, we now proceed to his posthumous works.

The first part contains experiments to support an opinion of Dr. Marshal, that no fluid whatever is found in the pericardium of an animal, unless occasioned by previous disease, or the manner of death. The experiments are few and decisive, but we cannot say important; for, whether that membrane contains a *tea-spoonful* of fluid, or only a condensation is found after death of a fluid constantly exhaling during life, scarcely deserves the sacrifice of the few victims which were made on the occasion. They are, however, worth recording, particularly that in two drowned cats a bloody fluid was found in the pericardium, and also in the pleura; and that no fluid was discovered in the ventricles of the brain.

Two cases of hydrophobia follow, related, no doubt, with equal accuracy and minuteness. Such histories have been now so multiplied, that they lose much of their interest, unless of recent occurrence. A comparative view of the symptoms follow; and, after that, the examination of each after death. This, we must say, is by far the most important.

"The body of the lad," says Dr. M. "which I examined by the direction of Dr. Pitcairn, in the public theatre of the hospital, before many witnesses, all competent to judge of the facts presented, and about a day and half after his death, a little after midsummer, I found as follows:—

"The whole cavity of the mouth, and internal fauces, was superficially whiter than usual, and made wet with a whitish thin saliva, standing frothy, as if in a lather, in all the natural interstices about the isthmus of the fauces. The membrane, lining the whole cavity of the mouth and internal fauces, was unusually firm, and contracted tight around the tonsils, which stood out round, hard, white, and smooth, like two buttons;—the velum pendulum palati was contracted in breadth, and the uvula appeared firmer and rounder than common.

"The passages up into the nose were not particularly searched; the passages downwards were altered somewhat in capacity, and in the structure of the parts:—the pharynx was thrown into longitudinal wrinkles, and the same appearance was found in the œsophagus. The top of the larynx was unusually open, and the epiglottis appeared a little narrower, and more peaked than usual;—the ligaments of the glottis appeared to be unusually tense. Whether the wind-pipe downwards was contracted, did not then occur as a matter to be looked into. The membrane of the mouth and internal fauces, as well as the inside of the larynx, trachea, and œsophagus, were marked here and there with dark purple-like suffusions, and, in some places, coloured with distinct ramifications of small distended red vessels. The dark purple-like suffusions appearing also about the upper parts of the pharynx; a scarlet uniform redness over the whole tongue, at the roots of the paler papillæ. There were ramifications of red vessels about the epiglottis, and inside of the larynx; and the inside of the trachea was coloured with vessels of a darker red.

"When the chest was opened, we found the heart rather of an under size for the body; the anterior ventricle flat; the sinistro-posterior ventricle rounded, but unusually firm, even approaching to hardness. The ventricle was empty, and the substance of the whole heart, when cut into, was pale in colour, and, as said of the left side, unusually firm; a dark suffused spot or two appearing under the investing membrane, near the basis of the sinistro-posterior ventricle, as if from the rupture of some minute vessels in the substance of the muscle.

"The great vessels of the thorax appeared, with the vasa vasorum, as if minutely injected, and of a vermilion colour, especially the great oblique arch of the aorta. The pericardium was slightly reddened with ramifications of the vessels. The whole pleura of the left lung of the same vermilion red colour, with turgid vessels. When this lung was cut into, some liquid blood of the same light red colour oozed out of its substance.

"Some adhesions, evidently the effects of a former affection about the breast, having nothing of the gloss of the surfaces mentioned, tioned, were found between the left lung and ribs. Right lung was not so reddened; but the dark red appearance of the inner membrane of the trachea descended into the bronchia, and entered into the internal surface of both lungs.

"No other traces of morbid alteration were noticed in the thoracic viscera. The lungs were perfectly free from tubercles, soft, spongy, and uniform in their texture.

"The abdomen presented no less conspicuous, and, I think, somewhat similar morbid alterations. The stomach, otherwise perfectly sound in all its texture, was, however, marked in the inside with broad purple-like suffusions, especially about its thick end; the natural rugæ deeper than usual, and nearly empty: it also seemed to me to have taken on a morbid degree of elasticity, for, on expanding the rugæ, by extending a portion of it, it returned with unusual force to its wrinkled state.

"The pylorus appeared like a narrow ring projecting upwards into the cavity of the stomach, with scattered ramifications of distended red vessels, in the omentum and mesentery.

"The inmost coat of the intestines was marked in different places with dark purple suffused spots; the intestinum ilium was full of rugæ; and the rugæ of the villous coat of the rectum were deep, and larger than common. This intestine being expanded, resisted, and returned with an elasticity, like that of the stomach. Soft fœculent matter of the usual colour remained in the great intestines.

"The liver was of the usual healthy appearance, but the ductus cysticus was constricted, and almost impervious; whether there was any unusual construction of the ductus communis, was not adverted to.

"The tunica propria of the kidneys was coloured with numerous ramifications of blood-vessels, containing a blackish blood; their substance was whitish, and in the infundibula a whitish gruel-like fluid was found.

"The bladder was contracted, quite empty, and marked in some places with dark suffused spots. The urethra, being divided at the membranous part, was contracted to the size of a corking pin.

"Last of all the head was opened, and the following appearances were observed. An unusual moisture and lubricity between dura mater and arachnoidea; and the pia matral vessels appeared more numerous, blacker, and more distended than is usual in health. When the substance of the brain was sliced off, not only were numerous reddish points left on the cut surfaces, but in a few seconds dark-coloured blood issued copiously from the cut vessels, and each point was converted into a broad blot of dark brown blood. The substance of the brain was in a small degree firmer than a healthy brain is; the ventricles of the brain contained a quantity of water, though they were not distended with it, and on its being let out, the fine membrane lining the ventricles was seen marked with ramifications of vessels, turgid with dark-coloured blood.

"*Morbid*

" *Morbid Appearances in the Woman.*—Dr. Hamilton, when he was informed that the appearances above-mentioned had been observed in the body of the lad, with a liberal spirit of medical research, caused the body of the young woman to be retained for a similar examination. The dissection was accurately performed by Mr. Blizard, (now Sir Wm. Blizard,) in the operating theatre of the hospital, before a number of competent judges of morbid appearances.

" The body had been dead about a day and a half, in temperate weather of the month of April, and no visible putrefactive change had commenced.

" The head was first opened, and the following morbid alterations appeared. The dura mater, instead of having the healthy colour, was of a dark hue; the larger branches of its arteries, which in health are the only red arteries visible, were tense and turgid, with livid liquid blood; and the smaller branches, which are usually colourless, were filled, as if strongly injected, with blood of the same colour; the veins were distended with the same kind of blood. As a membrane, it was unusually dark-coloured, from being overloaded with blood; and being lacerated in one place in the separation, upwards of a tea-spoonful of the same livid coloured blood was shed on the outside of it.

" There was water between the dura mater and tunica arachnoidea, so copious, that when the brain was drawn aside against the lower part of the skull, it collected in a considerable quantity between the brain and dura mater, at the edge of the divided skull.

" Water was also evident between the tunica arachnoidea and pia mater, for the cellular membrane which connects the two was swollen with it. There was also water between the pia mater and brain. When the brain was cut off in slices, the surfaces showed numerous brown points, which quickly spread into as many blots of the same colour, larger than any I had ever seen in any brain before.

" Both lateral ventricles were distended with a straw-coloured fluid; and the same kind of fluid was found in the other ventricles, with a watery appearance of the plexus choroides.

" The basis of the brain presented an appearance altogether new to me. The four arteries of the brain were tense, and filled with the same kind of livid brown blood, and all their branches in the pia mater were much distended with the same kind of blood.

" In the neck the jugular veins were unusually small, but very tense and full; the carotid arteries were also tense and small.

" In the thorax, the heart was found situated unusually erect; left side unusually firm, and containing some liquid dark brown blood; right side of the heart flaccid and empty. The oblique arch of the aorta smaller than usual, and contained a florid coagulum.

" The whole membrane lining the larynx (mouth not examined) was marked with dark red suffusions; and the membrane of the trachea downwards into the lungs was of an uniform red colour.

 " The

"The surface of the lungs or pleura was of a dark red colour, with numerous suffused patches of a more florid appearance.

"Dark patches, or suffusions of a dark colour, appeared on the inside of the stomach, and here and there in the small intestines. Stomach empty, and firm in its texture. Pylorus unusually projecting into the cavity of the duodenum.

"The contents of the pelvis were coloured with uniform suffusions, or purple-coloured ramifications of turgid blood-vessels. Bladder of urine was marked inside with dark suffusions, and almost empty."

Some remarks follow "on the pathology of hydrophobia," which, to say the least of them, are as good as any others that have been offered.

An appendix to this account is added, by Mr. Sawrey, containing some further cases, with judicious remarks.

Part the 3d is on the Morbid Anatomy of the Brain in Mania. This is the result of several examinations in Bethlem Hospital. They are divided into two classes. In the first, the brain only was examined; in the second, the heart and arteries. Such records are always valuable. Mr. Sawrey, in his introduction, remarks, that, before these examinations, a general opinion prevailed that no morbid appearances were to be detected in the brain of maniacs. We venture to differ from him. Before Dr. Marshal was known in London, an opinion prevailed that the brain of maniacs was particularly firm. Dr. Baillie refers to this opinion in his Morbid Anatomy, but considers it as shaken by Mr. Haslam's observation. We confess, however, we are more disposed to attend to the remarks of a practical anatomist; and it is certain that the exceptions to this state of the brain are very few in the histories before us. The records are rich, and extremely valuable. We only wish they were still more numerous. In the first were discovered, among other appearances, air bubbles in the veins at the basis of the skull,—a fact which has been denied by many, and observed by very few. In most of the others, the brain was particularly firm, the ventricles enlarged, and filled with fluid; the veins of the pia mater in many turgid, with dark-coloured blood; the tunica arachnoidea either suffused with a gelatinous substance, or filled with fluid limpid or straw-coloured. In one, this appearance, with the hardness of the brain, was attended with a peculiar deficiency of blood in all the vessels about, and even in, the substance of the brain; ossification of the arteries was frequent. There are fifteen cases in which the brain only was examined. Seven follow in which the heart and arteries were examined also. Whether this difference arose from some morbid symptoms during life, we are not informed; nor by the previous history, can such symptoms be always inferred. It is, however, remarkable that none of the subjects were without some diseased appearances about the thorax, which shows the propriety, in all cases, of examining all the cavities.

The fourth part contains Observations on the Nature of Mania. An introduction informs us that the remarks were penned at different times by Dr. Marshal, and are now collected by the editor. After this, we have a long chapter on the functions of the brain and nerves. This is replete with matter partly physiological, pathological, and metaphysical. We cannot condemn our author if he has offered nothing new or satisfactory on these intricate subjects; but we regret the time which men of industry and integrity lose in this way. There are, however, remarks on the effect produced on the brain, and thence on the mind, by generally disturbed vascular action, which, to say the least, repay the labour of perusing the chapter.

The second chapter is on the proximate Cause of Mania. These the author refers to altered action in the vessels of the brain, or of the arterial system; and certainly the dissections, with the histories by which his work is enriched, seem to justify the conclusion. But still there is a difficulty which the author only slightly hints at. If he has no more knowledge on the subject than he gives us, he should have acknowledged it; if he has, it should have been communicated. We refer to his remarks on the original formation of the brain, and the effect produced by it on the intellect of the subject. We can scarcely doubt the truth of this; but, when Dr. M. speaks of it as demonstrable, we cannot but feel a wish to know his facts.

A chapter follows to account why "marks of disease are not always apparent in the brain of maniacs after death." Here the author considers that there may be so strong a predisposition or liability to the madness, as may be sufficient to induce the disease from very slight causes, and that, when the disease is induced, an action must take place in the brain, which may become permanent or be re-excited from similar causes. We are not disposed to doubt this; but does it prove more than that in such subject there is a peculiar conformation of the brain, which hitherto has not been discovered? If discovered, we shall probably not be able to alter it; but we may learn to make certain provisions in such subjects, as will be the means of exciting as little as possible such a latent disposition into action. This subject is somewhat illustrated in a short chapter "on the predisposing or exciting causes." The last chapter, "on the varieties rieties of madness," cannot be expected to contain much information.

On the whole, however, we may regret that the work is not so complete as we could wish, we are ready to admit that it contains much information. We sincerely hope that the liberal example here given of nearly thirty cases of dissection of maniacs who died in a public hospital, and whose bodies were examined by a professed anatomist, will be reduced to a register; and that by such an accumulation of facts, something may be discovered concerning this formidable and melancholy complaint.

---

*Edinburgh Medical and Surgical Journal*, October, 1814.

The following are the remaining papers of No. XL. not yet noticed in our Journal.

*Report on the Use of Charcoal Powder as a substitute for Cinchona.* By R. Calvert, Physician to the Forces.

So many remedies are well known in the cure of intermittents, that there is reason to believe we sometimes forget the disease was ever cured before the introduction of the Peruvian bark into Europe. The snuff of a candle in wine is an old remedy all over Italy. We mean not by this to undervalue the hint here offered, as it is impossible to multiply our resources too much, especially against diseases of frequent occurrence, and sometimes untractable by medicines of known virtue.

*Case of Singular Affection of the Mamma, cured by the use of Iron and Kali Purum.* By Thomas Salter, Esq. Member of the Royal College of Surgeons, London.

This disease seems to have been that species of cancer which Dr. Adams denominates *Hydatis cruenta*, with this peculiarity, that the cysts were on the surface. It will be recollected, that from the theory Dr. Adams built on the separate vitality of those cysts, Mr. Carmichael attempted the cure of them by the use of iron, inferring, that as all aquatic animals are injured by ferruginous springs, the cysts, if really animalcular, might suffer in a similar manner. His success has produced a very general use of the remedy. The present paper illustrates the theory as far as can be judged by general analogy. The life of the cysts appears to have been destroyed; but to dislodge their dead tunics, which adhered in the form of sloughs, a caustic application was found necessary. The carbonate of iron was also used internally.

*Case of Ischuria Renalis.* By Dr. Laing, Fochabers.

This case exactly resembles others of inflamed kidneys, and gave way to the antiphlogistic treatment. The author remarks,

"In this case, the functions of the kidneys appear to have been entirely suspended for nine or ten days, without any material injury resulting to the rest of the system. During this period, the perspiration was not sensibly increased; and there was none of that urinous smell about the patient which frequently attends suppression of urine. The only evident outlet for the quantity of liquids introduced into the system, was the copious watery discharge produced by the purgatives. The immediate cause of these symptoms appears to have been an inflammatory affection of the kidneys, indicated by the pain in the back, feverish symptoms, and sizy blood; but differing materially from ordinary nephritis, in the absence of the acute pain, high fever, vomiting, and frequent painful micturition, which characterize that disease, and in the total suspension of the urinary secretion. On what this difference depended, it is not easy to determine. Probably the membranous coverings of the kidney may be principally affected in acute nephritis, and its medullary substance in the present case. This would, in some measure, account for the more acute pain, fever, and vomiting, in the former case; and for the less urgent general symptoms, but more complete suspension of the functions of the organ, in the latter."

There are certainly different degrees of inflammation, but a suspension of the secretory function prevails more or less in all; nor should we be quite certain, where a patient makes many watery stools, that no urine is ever mixed with them.

*Remarks on the Puriform Ophthalmia.* By William James Wilson, Fellow of the Royal College of Surgeons, London, and Surgeon at Manchester.

This is a good practical paper, but contains nothing very new.

*Observations on the Use of Ipecacuanha and Opium in Dysentery.* By Mr. William English, Surgeon.

The use of ipecacuanha in small doses, combined with opium, is well known in the chronic dysentery. Mr. English wishes to recommend, in a more severe form of the disease, large doses, to the quantity of 20 or 30 grains, given with a still larger dose of tinct. opii. By these means the ipecacuanha will sometimes remain on the stomach altogether; at others will not be vomited till several hours after it is taken, and in both cases produce very beneficial effects. If the medicine is ejected as soon as swallowed, it is always repeated, repeated. The cases given are pointed, and the whole paper may be considered as useful and practical.

*Observations on the necessity of Opening the Sac in the Operation for Hernia.* By J. PENKIVIL, Surgeon, Plymouth Dock.

This is a well-digested paper, and shows, with much propriety, the absurdity of the great apprehension at one time entertained of admitting the external air within the abdominal cavity; and also the importance of opening the sac in most operations for hernia.

---

### *Medico-Chirurgical Transactions, Volume the Fifth.*

*(Continued from p. 69.)*

DR. CHESTON now determined to remove the bony case, and raise the head from the chest. The left arm and thigh, as well as the parts of generation, were destroyed in the investigation; but the right arm and leg were brought into view by a careful removal of the left foot and right side of the face.

"The position of the child in the cyst was very similar to that which it holds in the uterus; in which the body and limbs are brought nearly into a globular form. The spine was incurvated; the head bent forwards upon the chest, and the pelvis upon the abdomen, and the limbs folded between the pelvis and head. All the parts were most forcibly compressed, and, as it were, closely squeezed together by the bony cyst; hence the limbs were all distorted and deformed, and the figure of every part variously affected. Towards the middle of the tumour, the body and limbs, when carefully separated, were found in the most complete state of preservation: the skin, adipous substance, and muscles, retained much of their natural consistence and characteristic appearances; but parts were less distinct on the circumference, from the strong adhesion of the bony covering to the whole surface of the mass. The cyst grew so firmly to the child, that it could only be separated by very forcible means; by the employment of which the soft parts attached to the internal surface of the tumour were necessarily sacrificed, and they presented a very imperfect and mutilated appearance when this separation was accomplished. It seemed that the cyst had absorbed the integuments and muscles of the parts which were situated in contact with it; thus, in one of the arms, which occupied this position, I found that half of the limb, which was turned towards the centre, as full and plump as usual, while the other portion lying towards the circumference, had lost all the soft parts, down to the bone, which was in contact with the cyst, and firmly compressed by it through its whole ex-

 tent.

tent. The scalp, on which there were some trifling remains of hair, had lost its firmness and consistence, so as to separate from the cranium on the slightest touch. The integuments of the face, body, and limbs, still retained so much of their natural plumpness, that, by the compression, the contiguous parts were reciprocally indented.

"The contents of the thorax and abdomen, as far as their exposure by the longitudinal section would enable me to judge, retained much of their natural appearance, and not the least tendency to putrefaction could be observed in any of them.

"The brain was rather more firm than in its recent condition, and nearly of its natural colour. The lungs were in a compact state. The liver was of a dark brown or umber colour, and the intestines deviated but little from their usual membraneous appearance, though compressed together into an irregular mass.

"Although the body of the child was in a very complete preservation, and all its component parts could be readily distinguished, yet it exhibited a very different appearance from that of a subject lately deceased. It was very firm and compact, as if condensed by great pressure; hence it seemed completely deprived of its fluids, and formed, therefore, a contrast to the juicy state of a body recently deprived of life; of blood, either in a fluid or coagulated state, (with an exception that I shall notice directly) or of that colour, which results from this fluid being contained in the vessels of a part, there was not the slightest appearance. The muscles, instead of being of a bright red, and fleshy, were of a brown hue, and the integuments possessed a very light brown or yellowish tint; the colour in both cases was somewhat like that produced by long immersion in spirits of wine. The bones were brown, and drier than usual; they separated very easily from the periosteum and epiphyses. Of the membranes, placenta, or navel-string, I could not discover any remains, excepting the insertion of the latter into the body of the child.

"One circumstance more deserves to be noticed: near the upper part of the cyst, internally, was a coagulum of blood, apparently in as recent a state as if it had not been extravasated from the vessel an hour before; it was full in colour, and firm in its substance, but, on exposure to the air, it soon dried, leaving little more than a mere stain on the part."

The author is decidedly of opinion that the child was originally contained in the uterus, and remained there the usual term of gestation.—He concludes this very interesting narrative with some ingenious remarks, and the following singular case.

"Jane Hawes, a healthy well-made woman, about twenty-five years of age, was the mother of two very fine children. Her labours had taken place at the proper periods, and were natural in every respect. The last child was born after a very quick labour; and and the mother, in each instance, got well in a short time, without the occurrence of any thing worthy of remark.

"In the summer of 1795, when her youngest child was about two years old, she became again pregnant, and I was desired to attend her. She went her full time without the smallest inconvenience, and, at the end of nine months, was seized with pain, which was supposed to be labour, and I was sent for. When I entered the room, the pain appeared to me to be so acute, that I concluded her delivery would shortly take place, as had happened in the former instances.

"Upon examination, however, *per vaginam*, I was surprised to find no disposition to dilatation of the os tincæ. The neck of the uterus was quite obliterated, and I could plainly feel the child through its substance; but though the pain was strong, there did not appear to be any forcing power accompanying it, and it had not that peculiar striving on the part of the mother, so familiar to those who have been in the habit of attending cases of midwifery. As she had no disposition to fever, and felt no inconvenience during the remission of her pains, I contented myself with directing an anodyne medicine, and thought it most prudent to wait patiently, and observe what course the efforts of nature would take.

"These pains, however, continued for nearly three days, during which time no further advances were made to delivery; and they were, from time to time, mitigated by opium and other antispasmodic remedies, with fomentations applied to the whole region of the abdomen.

"During this period, I had frequent opportunities of satisfying myself that the uterus had undergone that change which an ordinary pregnancy produces; and I could not but conclude, that at this time the child was in its cavity. In the beginning there had been a small discharge, which increased on the third day in quantity, and became coloured with blood.

"On the third day, fever, and symptoms of inflammation took place; and these spasmodic pains, which had at times been very violent, by degrees subsided, and were succeeded by tenderness of the belly, and pain arising evidently from a new condition of parts. These symptoms were controuled by an antiphlogistic plan; and in the course of ten days, the inflammatory symptoms began to subside, and were succeeded by considerable discharges of a very fœtid and purulent matter.

"In the progress of this case, during the following six months, great emaciation, with hectic fever, came on, and her strength was supported, as well as it could be, by bark, wine, opiates, and other tonic medicines, together with as generous a diet as her stomach was capable of bearing. About this time the discharge began to diminish, and her general health improved; and in about fifteen months from the beginning it had entirely ceased, and she regained her usual flesh and strength.

"During this time, her size had gradually diminished; and she was was left with an enlargement of her belly, about equal to the size of a woman six months gone with child. She resumed her usual occupation, which was that of keeping a grocer's shop, and continued perfectly well till the autumn of the year 1800, when, to my great surprise, she told me she suspected she had again become pregnant.

"Upon inquiry, I found that menstruation had regularly taken place for more than a year before this time; and, upon hearing all she had to say, I was satisfied that her conjecture concerning herself was well-founded. She went on without any thing remarkable happening, till the December of that year, when she was taken regularly in labour.

"Upon examination, I found a round solid substance, occupying the upper part of the pelvis, and extending from the base of the sacrum, to within less than three inches of the upper rim of the pubis.

"At first I was deceived, and took this substance for the head of the child; but a more minute examination convinced me that it was a solid bony tumour, immoveable, appearing to be firmly fixed to the base of the os sacrum. On passing my fingers as high as I could, between the anterior surface of this tumour, and the pubis, I plainly discovered the os tincæ, in a state of dilatation; and I could distinctly feel the foot of the child.* As her pains were not very rigorous, and her strength and spirits were unexhausted, I waited, and allowed the labour to take its usual course. It was evident, however, that there was not room enough to save the child, and at a proper time I proceeded to deliver her by the feet.

"This proved to be a work of great difficulty, and it was impossible to succeed without diminishing the size of the head by the crotchet.

"The whole of the child was brought away, and the usual discharges took place. I apprehend that some violence might have been done to this bony tumour, which was so much in the way; for symptoms of very extensive inflammation came on, shortly after her delivery. Suppuration followed, with immense discharges of fœtid matter, with hectic fever, and great emaciation. She lingered on in this wretched state, till ulceration took place, which extending downwards, in the course of a few months, destroyed

"* At this time I was desired to see her with Dr. Briggs and Mr. Newell. The cæsarian operation was mentioned, but rejected from the too probable diseased state of neighbouring parts. It was, therefore, determined to quiet general irritation by opiates, and to guard against any inflammatory tendency in the uterus by cold spirituous applications over the abdomen. These means were pursued for two days with every apparent success; and on the third, Mr. Newell, finding some further advances towards the exclusion of the child, proceeded to give her every assistance."

the the vagina and rectum, and her stools, urine, and discharges, came, seemingly, out of one great cavity. During the progress of this wretched case, various bones of the former fœtus were discharged, and, after lingering in the most cruel sufferings for about ten months, the unfortunate woman died.

"It is very much to be lamented, that no entreaties could prevail with her friends to allow an examination of the body. The fact of the reality of the former impregnation was sufficiently proved by the discharge of fœtal bones; but dissection would doubtless have thrown much light upon this very singular case."

An account of some Diseases of the Toes and Fingers, by Mr. James Wardrop, contains some good instructions for remedying defects in those parts, which, from slight and inconsiderable beginnings, are ultimately productive of great pain and serious distress. The first affection which he notices is where inflammation and suppuration take place in the soft parts contiguous to the nail, generally denominated "the growth of the nail into the flesh." The disease, however, does not originate from any peculiarity in the growth or formation of the nail, as has been hitherto supposed, but, according to Mr. Wardrop, by its mechanical resistance to the tender flesh, occasioning a constant source of irritation. The cure consists in applying caustic to the soft parts.

Ulceration at the root of the nail sometimes produces very unpleasant consequences, as the loss of a nail, and even of a limb. Mr. Wardrop has found benefit in these cases by a course of mercury.

For corns, he recommends, as certain remedies, the application of caustics, or of a strong solution of muriate of mercury, after cutting off as much of the corn as possible. In chilblains, he warmly recommends an embrocation of six parts of soap liniment to one of tincture of cantharides.

Mr. Wardrop is very commendable in bestowing his attention upon these subjects, which are often shamefully neglected by men of superior education; and we think him liberal in publishing the result of his practice.

In a paper by Dr. Stewart, upon some of the Causes which destroy the Fœtus in Utero, an interesting case is related. The patient, it seems, was delivered of a dead fœtus for eight successive times, in spite of the remedies which Dr. Stewart employed. The chief symptoms which he had to combat, occurred in the sixth month of pregnancy, and were severe pains in both sides, and in the abdomen, accompanied by diarrhœa. They continued till the middle of the seventh month, when they suddenly remitted.

In the ninth pregnancy, Mr. Croft was consulted. He directed, "that, in addition to the use of the cold shower-bath and and tonics combined with antispasmodics, as soon as symptoms of irritation of the bowels came on, two or three grains of opium should be introduced into the rectum, and that this was to be repeated as often as there was any degree of tenesmus." The patient found so much relief from this plan, that she went on to the full period of pregnancy, and was delivered of a healthy boy.

Mr. Lawrence, of St. Bartholomew's Hospital, has furnished an account of a Child born without a Brain, and which lived four days:

"The brain and cranium were deficient, and the basis of the latter was covered by the common integuments, except over the foramen magnum, where there existed a soft tumour, about equal in size to the end of the thumb. The smooth membrane covering this was connected at its circumference to the skin. The child, as is generally the case in such instances, was perfectly formed in all its other parts, and had attained its full size. It moved briskly at first, but remained quiet afterwards, except when the tumour was pressed, which occasioned general convulsions. It breathed naturally, and was not observed to be deficient in warmth, until its powers declined. I regret, that from a fear of alarming the mother, no attempt was made to see whether it would take the breast; a little food was given to it by the hand. It voided urine twice in the first day, and once a day afterwards: it had three dark-coloured evacuations. The medulla spinalis was continued for about an inch above the foramen magnum, swelling out into a small bulb, which formed the soft tumour on the basis of the skull. All the nerves, from the fifth to the ninth, were connected to this. The intestines contained a moderate quantity of the usual dark-coloured substance; and there was a little fluid, of the ordinary appearance, in the gall-bladder. Sœmmerring and Morgagni have observed, that most of these acephalous children are females; and it has been found, in many instances, that the renal capsules were very small. The present case exemplified both these observations."

In a second case of malformation which Mr. Lawrence witnessed, the whole of the spinous processes were deficient, and the place of the medulla spinalis was supplied by a vascular membrane, like that which covers the basis cranii in acephalous children, united in the same way to the surrounding skin. The heart, lungs, and liver were deficient; and the ribs, short and imperfect, lay close to each other, and did not form a thoracic cavity. The face was malformed. The fingers and toes were fewer than usual: in other respects, the formation of the body and the size of the limbs were natural. Having stated these cases, the author gratifies the admirers of human curiosities with a choice selection from various writers, ancient and modern; we have histories of spotted men, porcupine men, men with all the internal organs transposed, supposed hermaphrodites, double bodies, double heads, bladderless beings, imperforate rectum, one-eyed men, and men whose heads do grow beneath their shoulders: in short, the assemblage of monsters is rare and extensive. If the catalogue furnished by Licetus contain a greater number and variety of specimens than the selection of Mr. Lawrence, the latter has the advantage in his instances being supported by indubitable evidence. Hitherto such cases have been of no other utility than to prove that nature is not always perfect in her works; while the reasoning upon them has been vague and ridiculous. Aristotle, indeed, has usually been resorted to for the solution of all knotty points in the mysteries of nature, and the adepts in his philosophy have no difficulty in explaining the origin, cause, and final design of monstrous productions. We need hardly observe, that Mr. Lawrence has not contented himself with merely compiling a volume of facts in themselves very useless; he has refuted various absurd opinions which have prevailed upon this branch of natural history; and his whole paper is interspersed with original observations and just deductions, some of which we shall transcribe; and we have the greater satisfaction in so doing, because we know of nothing tolerable on the subject in any other work.

"Notwithstanding the general similarity of parts in the same species of animals, there is considerable variation in those details of structure which do not affect the execution of the functions, nor interfere with the general form and relations of organs. The smaller parts, and particularly the blood-vessels, differ in almost every two bodies; so that it would be very difficult, if we descended into minutiæ, to settle precisely what ought to be regarded as the most frequent, and therefore the *natural* structure. In parts, however, where one model is generally adhered to, deviations occasionally take place: these aberrations from the accustomed type, are called by anatomists *varieties*, or *lusus naturæ;* when the body in general, or some large and conspicuous part of it, deviates from the accustomed formation, which deviation is accompanied generally with imperfection in some of the functions, the creature is called a *monster.* No very accurate line can be drawn between these and varieties; nor can we assign a rigorous meaning to the former term, which is generally used in a loose and popular manner.* A considerable anomaly in the form or structure of a particular organ is often called by anatomists a *monstrous* formation.

"* 'Monstri vox,' says Haller, 'ex ipsa linguæ natura videtur designare aberrationem animalis a consueta suæ speciei fabrica adeo evidentem, ut etiam ignarorum oculos feriat. Nobis vis vocis perinde videtur indicare fabricam, etiam grandium & conspicuarum partium, alienam a solita.' Oper. Minor. tom. iii. p. 3."

"In the articular ends of bones, there is little variety: a particular shape is better adapted to a particular kind of motion: but in other parts, as the foramina, depressions, ridges, and sutures, deviations from the accustomed model are often observed. The same general rule will apply to the varieties of muscles: the principal object is a certain insertion near a joint, giving a determined direction to the motion produced. These insertions vary very little; but there are many differences in other points, which have no share in regulating the motion. The biceps flexor cubiti has often an additional slip from the humerus, and the latissimus dorsi from the angle of the scapula. The palmaris longus and the plantaris are often absent; but the other flexors of the wrist and extensors of the ankle supply their place.

"In no part of the body are the arrangements less confined to a particular model, than in the distribution of the blood-vessels. Whether the blood pass by one route or another, is of no importance. The great arterial trunks of the body and limbs are not exposed to these varieties, because they generally occupy situations in which they are most effectually protected from external injury. We may remark, also, that the arteries of the upper are much more liable to varieties than those of the lower limbs. The latter are almost constant in their distribution, while those of the fore-arm and hand are hardly alike in any two subjects taken together. There is no obvious principle by which this difference can be accounted for. One or two examples have been observed, in the vena portarum, of a departure from the usual arrangement, completely deciding an important physiological question, which it had not been possible to settle by direct experiment. The trunk of this vein, instead of branching out in the liver, has terminated in the inferior vena cava. Mr. Abernethy found this in a child about ten months old, in which the gall-bladder contained bile, and the body had been well nourished. Another instance, not yet published, was met with by a teacher of anatomy in London, in an individual several years old. As the blood, which had circulated through the digestive organs, passed immediately into the general venous system in these cases, the bile must have been secreted from the blood of the hepatic artery; although so many ingenious physiologists have proved, quite to their own satisfaction, that the blood acquires in its circulation through the intestines, omenta, and spleen, various properties which are indispensibly necessary to the formation of bile.

"There is less variety in the nervous system of animals of the same species, than in most parts of the body. Scarcely any differences are observed in the appearances of the brain, and much fewer in the distribution of the nerves, than of the blood-vessels.

"There is very little variety in the organs of sense; perhaps the mechanism of these, and of the brain and nerves, is nicer, so that a considerable deviation from the ordinary structure would interfere with their peculiar functions.

"Irregularities in the organization of the skin, are more conspicuous spicuous in the coloured than in the white races of mankind. One of the most striking is the entire absence of colouring matter, constituting the albino, which was first noticed in the negro: this peculiar formation, however, occurs also in the white races, and in various genera, both of mammalia and birds. The colouring matter is equally deficient in the hair and eyes: hence the former is white, and the choroid coat, iris, and pupil of the latter pink."

After brushing off the silly notions of some old women, and of certain ingenious physiologists that would attribute the nævi materni, and many unnatural malformations, to the agency of the mother's imagination on the fœtus in utero, and making some pointed remarks on the effects of domestication in producing disease in animals, Mr. Lawrence proceeds:—

The circumstances just mentioned; the great abundance and numerous kinds of monsters found in the human subject, their comparative rareness and fewer species in the domestic animals, and their entire absence in the wild races, lead us to suspect that they owe their origin to something connected with our peculiar mode of existence in this respect; in short they resemble our diseases, which I believe to be altogether unknown to animals in a state of nature, and to exist in greater number, in proportion as they are more and more completely domesticated. At the top of the scale, whether we regard the number, the complication, or the severity of his diseases, stands the lord of the creation; he has in truth created arts and sciences, made wonderful progress in knowledge and civilization, and he may boast that he has subdued both animate and inanimate nature; but the long and appalling catalogue of the nosologist is well calculated to check his triumph, and make him doubt whether he has not paid too dear a price for empire.

Generation is a function not differing in its essential characters from the other processes of the animal economy. The production of a new being seems on a superficial view so much like creation, according to the notions which men have amused themselves with framing on that subject, that they have conceived it to require some preternatural agency. Regarding this business then as the work of God, and having already assumed that all his works are perfect, they maintain that the young animal is originally perfect, and degenerates into a monster through the action of external forces. More accurate observation discloses to us in this affair merely the operation of secondary causes, and exhibits to us the production and development of the fœtus, as the result of vascular action in secretion and nutrition: in short, however his pride may be offended at hearing it, the simple truth is, that man, considered at the epocha of his first formation, is merely a secretion from the vessels of the ovary.

The function of generation is not more exempt from the operation of disturbing causes than any other in the animal economy. Any violent and sudden impression interrupts it at once by causing abortion; but minor causes, although their effects are not seen, are not to be deemed inoperative. Particular bodily formations, particular mental characters, and dispositions to certain diseases, &c. are transmitted to the offspring. Indeed, how can we expect that when all the rest of the being is artificial and vitiated, this one part should be undisturbed? I ascribe then the aberrations from the usual form and structure of the body which constitute monsters, to irregular operation of the powers concerned in generation, and place them on a level with respect to their cause, with unhealthy executions of the nutritive, secretory, and exhalant functions. I only mean by these observations, to refer the aberrations of the formative process to the same general principles as the other deviations from the healthy execution of functions, and to protest against the considering them as forming a peculiar case out of the common rules applying to organized beings.

Mr. L. considers the cases of creatures contained in the bodies of others as double fœtuses, or as examples of additional parts annexed to a natural body, in consequence of original malformations.

From the facts now adduced, it results that cartilage, bone, ligament, cellular substance, membrane, intestine can be formed, where no brain, or nerve, or heart, exists, and where there is nothing further than the action of the vascular system; thus proving, that the brain, nerves, and heart, have no share in the formation and nutrition of our organs. It also appears, that the whole growth and formation of a fœtal body depend on the actions of the vascular apparatus. The facts stated by our author, support the opinions of Mr. Brodie and Dr. Le Gallois, that the action of the heart is independent of the brain, but is produced by the influence of the medulla spinalis. One of the acephalous cases also corroborates the fact ascertained by Dr. Le Gallois, that respiration does not depend on the whole brain but on a limited portion of the medulla oblongata, situated at a small distance from the occipital foramen and towards the origin of the pneumo-gastric, or nerves of the eighth pair. The important and interesting matter of this essay has induced us to extend our notice of it beyond the ordinary limits.

The remainder of the papers in these Transactions, being principally chirurgical, have been deferred, on account of some arrangements it was found necessary to make in that part of the editorship. The proprietors can now promise the assistance of a practical anatomist who has the range of an extensive hospital.

